# Supplementary material for: How children generalize novel nouns: An eye-tracking analysis of their generalization strategies
Source: PLoS One. 2024 Apr 3;19(4):e0296841. doi: 10.1371/journal.pone.0296841 (PMC10990231; doi:10.1371/journal.pone.0296841)
Supplement: S1 Table — (DOCX) [file pone.0296841.s001.docx]

S1 Table. Full list of materials.

Full list of materials for the Close vs. Far learning and the Near vs. Distant generalization conditions.

|  | Learning material | | | Generalization material | | | |
| --- | --- | --- | --- | --- | --- | --- | --- |
|  |  |  |  | Taxonomic items | | Distractors | |
|  | Standard learning item | Close learning item | Far learning item | Near | Distant | Perceptual | Theme related |
| Clothing accessories | *Bracelet 1* | *Bracelet 2* | *Watch* | *Jewel pendant* | *Bow tie* | *Tire* | *Hand* |
| Tools | *Hammer 1* | *Hammer 2* | *Axe* | *Pincers* | *Chainsaw* | *Ostrich head* | *Work room* |
|  |  |  |  |  |  |  |  |
| Food | *Apple 1* | *Apple 2* | *Cherry* | *Banana* | *Beefsteak* | *Christmas decoration* | *Knife* |
| Animals | *Ladybird 1* | *Ladybird 2* | *Beetle* | *Butterfly* | *Duck* | *Marble* | *Branche* |
| Music player | *Guitar 1* | *Guitar 2* | *Cello* | *Keyboard* | *Hi-Fi* | *Bottle* | *Music stand* |
| Game/toy | *Ball 1* | *Ball 2* | *Bouncing ball* | *Lego* | *Video game* | *Orange* | *Kids* |
| Food | *Pear 1* | *Pear 2* | *Strawberry* | *Pineapple* | *Fries* | *Candle* | *Basket* |
| Food 2 | *Potato 1* | *Potatoes* | *Endive* | *Carotte* | *Tarte* | *Rugby ball* | *Garden* |
| House tools | *Broom 1* | *Broom 2* | *Feather duster* | *Vacuum cleaner* | *Blender* | *Bush* | *Tiled floor* |
| Animals | *Snake 1* | *Snake 2* | *Lizard* | *Crocodile* | *Bird* | *Rope* | *Aquarium* |
| Vehicle | *Bike 1* | *Bike 2* | *Scooter* | *Rollerblades* | *Boat* | *Glaces* | *Helmet* |
| Office items | *Pencil 1* | *Pencil 2* | *Ruler* | *Scissors* | *Laptop* | *Candy* | *Hand* |
| Garden tools | *Spade 1* | *Spade 2* | *Rake* | *Pickaxe* | *Drill* | *Lolly pop* | *Garage* |
|  |  |  |  |  |  |  |  |
