# Supplementary material for: How children generalize novel nouns: An eye-tracking analysis of their generalization strategies
Source: PLoS One. 2024 Apr 3;19(4):e0296841. doi: 10.1371/journal.pone.0296841 (PMC10990231; doi:10.1371/journal.pone.0296841)
Supplement: S2 Table — (DOCX) [file pone.0296841.s002.docx]

S2 Table. Similarity ratings by category.

Perceptual conceptual and semantic similarity ratings by category

|  | Name | Conceptual distance ratings to learning items | | | | Perceptual similarity ratings to learning items | | | | | Semantic similarity ratings to learning items | | |
| --- | --- | --- | --- | --- | --- | --- | --- | --- | --- | --- | --- | --- | --- |
|  |  | Learning | | Generalization | | Learning | | Generalization | |  |  |  |  |
|  |  | Close | Far | Near | Distant | Close | Far | Near | Distant | Perceptually related distractor | Near | Distant | Thematically related distractor |
| 1 | Clothing accessories | 5.93 | 4.77 | 3.57 | 2.93 | 6.77 | 5.15 | 2.73 | 1.98 | 5.21 | 5.82 | 5.22 | 5.92 |
| 2 | Tools | 5.81 | 3.36 | 3.48 | 2.86 | 6.92 | 5.77 | 2.22 | 1.48 | 4.77 | 5.67 | 2.30 | 4.78 |
| 3 | Clothing | 5.67 | 4.15 | 4.13 | 2.89 | 6.92 | 5.15 | 1.60 | 1.10 | 4.33 | 3.33 | 2.57 | 4.67 |
| 4 | Food | 6.00 | 4.92 | 3.31 | 2.27 | 6.85 | 4.23 | 1.92 | 1.69 | 4.28 | 5 | 2.84 | 5.47 |
| 5 | Animals | 5.80 | 5.07 | 3.72 | 1.71 | 6.62 | 5.46 | 2.85 | 1.23 | 4.73 | 4.30 | 2.71 | 5.76 |
| 6 | Music player | 5.53 | 5.38 | 4.86 | 3.16 | 6.85 | 5.77 | 1.56 | 1.50 | 5.69 | 5.47 | 5.38 | 5.63 |
| 7 | Game/toy | 5.73 | 1.41 | 3.51 | 3.38 | 6.08 | 4.77 | 2.10 | 1.83 | 5.22 | 4.30 | 2.01 | 6.24 |
| 8 | Food | 5.46 | 4.93 | 4.44 | 2.59 | 6.69 | 5.31 | 2.10 | 1.75 | 4.52 | 5.67 | 2.27 | 4.78 |
| 9 | Food 2 | 5.56 | 4.87 | 4.11 | 1.92 | 6.23 | 5.23 | 1.96 | 2.28 | 5.42 | 4.37 | 1.5 | 6.00 |
| 10 | House tools | 5.23 | 4.73 | 3.69 | 0.79 | 6.77 | 5.23 | 1.85 | 2.30 | 5.42 | 5.67 | 2.27 | 4.78 |
| 11 | Animals | 4.92 | 4.87 | 3.67 | 2.58 | 5.92 | 4.08 | 1.60 | 1.79 | 4.75 | 2.63 | 2 | 5.47 |
| 12 | Vehicle | 4.62 | 5.13 | 3.08 | 2.88 | 6.69 | 5.08 | 2.06 | 1.70 | 4.08 | 5.00 | 2.06 | 5.89 |
| 13 | Office items | 5.38 | 3.50 | 3.24 | 1.70 | 6.46 | 3.62 | 1.47 | 1.47 | 4.40 | 5.06 | 2.75 | 5.92 |
| 14 | Garden tools | 5.54 | 5.13 | 2.64 | 4.13 | 6.85 | 4.62 | 3.75 | 3.94 | 3.96 | 5.25 | 5.28 | 5.77 |
