# Supplementary material for: How children generalize novel nouns: An eye-tracking analysis of their generalization strategies
Source: PLoS One. 2024 Apr 3;19(4):e0296841. doi: 10.1371/journal.pone.0296841 (PMC10990231; doi:10.1371/journal.pone.0296841)
Supplement: S3 Table — (DOCX) [file pone.0296841.s003.docx]

S4 Table. Results of Spearman correlations

Results of correlations between ratio Learn/Gen indicating children’s early attention to learning items rather than available options and their percentage of taxonomic answers.

|  |  | Near generalization | | Distant generalization | |
| --- | --- | --- | --- | --- | --- |
|  |  | Percentage of taxonomic answers | | Percentage of taxonomic answers | |
| Trials that lead to taxonomic answers | Ratio | r | *p* | r | *p* |
|  | Learn/Gen_Cor,1_ | 0.45 | < .001 | 0.58 | < .001 |
|  | Learn/Gen_Cor,2_ | 0.33 | < .001 | 0.32 | < .001 |
|  | Learn/Gen_Cor,3_ | 0.22 | < .01 | 0.36 | < .001 |
|  | Learn/Gen_Cor,4_ | 0.18 | < .05 | 0.17 | .06 |
|  | Learn/Gen_Cor,5_ | 0.31 | < .001 | 0.25 | < .01 |
