# Supplementary material for: How children generalize novel nouns: An eye-tracking analysis of their generalization strategies
Source: PLoS One. 2024 Apr 3;19(4):e0296841. doi: 10.1371/journal.pone.0296841 (PMC10990231; doi:10.1371/journal.pone.0296841)
Supplement: S4 Table — (DOCX) [file pone.0296841.s004.docx]

S5 Table. Details of the ANOVA run on M3 model.

Model includes Learn/Gen ratio for the first 3 gazes, in near generalization settings. Controlling for age and learning distance.

| Omnibus ANOVA Test on M3 | | | | | |
| --- | --- | --- | --- | --- | --- |
|  |  |  |  |  |  |
| Factor | Sum of Squares | *df* | Mean Square | *F* | *p* |
| Age | 13036.59 | 1 | 13036.59 | 23.84 | < .001 |
| Learning | 2.06 | 1 | 2.06 | 0.003 | 0.95 |
| Learn/Gen_Cor,near,1_ | 16546.84 | 1 | 16546.85 | 30.25 | < .001 |
| Learn/Gen_Cor,near,2_ | 5573.45 | 1 | 5573.45 | 10.19 | <.05 |
| Learn/Gen_Cor,near,3_ | 1575.56 | 1 | 1575.56 | 2.88 | 0.09 |
| Learn/Gen_Cor,near,5_ | 2996.17 | 1 | 2996.17 | 5.48 | <.05 |
| Residuals | 72731.34 | 133 | 546.85 |  |  |
