# Supplementary material for: How children generalize novel nouns: An eye-tracking analysis of their generalization strategies
Source: PLoS One. 2024 Apr 3;19(4):e0296841. doi: 10.1371/journal.pone.0296841 (PMC10990231; doi:10.1371/journal.pone.0296841)
Supplement: S5 Table — (DOCX) [file pone.0296841.s005.docx]

S6 Table. Details of the ANOVA run on M5 model.

Model includes Learn/Gen ratio for the first 5 gazes, in distant generalization settings. Controlling for age and learning distance.

| Omnibus ANOVA Test on M5 | | | | | |
| --- | --- | --- | --- | --- | --- |
|  |  |  |  |  |  |
| Factor | Sum of Squares | *df* | Mean Square | *F* | *p* |
| Age | 2982.97 | 1 | 2982.97 | 7.60 | .007 |
| Learning | 7.36 | 1 | 7.36 | 0.02 | 0.89 |
| Learn/Gen_Cor,distant,1_ | 15365.74 | 1 | 15365.74 | 39.09 | < .001 |
| Learn/Gen_Cor,distant,2_ | 1169.22 | 1 | 1169.22 | 2.97 | < .001 |
| Learn/Gen_Cor,distant,3_ | 5569.84 | 1 | 5569.84 | 14.17 | 0.035 |
| Learn/Gen_Cor,distant,4_ | 3157.31 | 1 | 3157.31 | 8.03 | <.05 |
| Learn/Gen_Cor,distant,5_ | 1065.38 | 1 | 1065.38 | 2.71 | .102 |
| Residuals | 47166.68 | 120 | 393.06 |  |  |
