# Supplementary material for: How children generalize novel nouns: An eye-tracking analysis of their generalization strategies
Source: PLoS One. 2024 Apr 3;19(4):e0296841. doi: 10.1371/journal.pone.0296841 (PMC10990231; doi:10.1371/journal.pone.0296841)
Supplement: S1 Data — (DOCX) [file pone.0296841.s006.docx]

S3 Supplementary results. 1.

Full results of the repeated measure analyses between all factors – including learning distance - for proportion of fixation times and log of the number of switches.

We performed a five-way repeated-measures ANOVA on the proportion of fixation times, with learning distance (close, far) and age (5, 6, 8) as between-subject factors and generalization distance (near, distant), time slice (beginning, middle, end) and AOI (L, Th, Ta, P) as within-subject factors.

Results revealed a simple effect of AOI *F*(3, 357) = 73.39, *p* < .001, $\eta_{P}^{2}$ = .38, interaction effects between generalization distance and time slice *F*(2, 238) = 3.20, *p* < .05, $\eta_{P}^{2}$ = .03, between time slice and AOI *F*(6, 714) = 86.01, *p* < .001, $\eta_{P}^{2}$ = .42. Results also revealed triple interaction effects between time slice, age and AOI *F*(12, 714) = 2.43, *p* < .01, $\eta_{P}^{2}$ = .04, and another between learning distance, time slice and AOI, *F*(6, 714) = 2.32, *p* < .05, $\eta_{P}^{2}$ = .02, and finally a four way interaction between learning distance, time slice, generalization distance and AOI, *F*(6, 714) = 2.12, *p* < .05, $\eta_{P}^{2}$ = .01.

A post hoc Tukey analysis with the interaction between learning distance, time slice and AOI revealed that there were no significant differences between learning conditions (See table S3.1). No significant differences were found between learning distance conditions in the interaction between learning distance, generalization distance, time slice and AOI.

Table S3.1: P-Values from a post hoc Tukey analysis on the interaction between learning distance, time slice and AOI.

|  |  | Time slice | | |
| --- | --- | --- | --- | --- |
|  |  | Beginning | Middle | End |
| AOI | L | *p* = 0.99 | *p* = 1.00 | *p* = 1.00 |
|  | Th | *p* = 1.00 | *p* = 1.00 | *p* = 1.00 |
|  | Ta | *p* = 1.00 | *p* = 0.83 | *p* = 1.00 |
|  | P | *p* = 0.86 | *p* = 0.99 | *p* = 1.00 |

We also performed a five-way repeated-measures ANOVA on the log of the number of switches, with learning distance (close, far) and age (5, 6, 8) as between-subject factors and generalization distance (near, distant), time slice (beginning, middle, end) and switch type (LL, LTh, LTa, LP, ThTaP) as within-subject factors.

Results revealed a simple effects of generalization *F*(1, 105) = 23,50, *p* < .001, $\eta_{P}^{2}$ = .18, time slice *F*(2, 210) = 75.32, *p* < .001, $\eta_{P}^{2}$ = .42, and switch type *F*(4, 420) = 40.75, *p* < .001, $\eta_{P}^{2}$ = .28 and two double interaction effects between time slice and learning distance *F*(2, 210) = 4.76, *p* = .01, $\eta_{P}^{2}$ = .04 and between time slice and switch type *F*(8, 840) = 11.47, *p* < .001, $\eta_{P}^{2}$ = .10.
