# Supplementary material for: How children generalize novel nouns: An eye-tracking analysis of their generalization strategies
Source: PLoS One. 2024 Apr 3;19(4):e0296841. doi: 10.1371/journal.pone.0296841 (PMC10990231; doi:10.1371/journal.pone.0296841)
Supplement: S2 Data — (DOCX) [file pone.0296841.s007.docx]

S7 Supplementary results. 2.

Full results of the repeated measure analyses between all factors and trial accuracy for proportion of fixation times and log of the number of switches.

We ran a four-way ANOVA on the proportion of fixation times from all trials from six year old children, with trial accuracy (correct, error), generalization distance (near, distant), time slice (beginning, middle, end) and AOI (L, Th, Ta, P) as within factors. Here, we focus on interactions with trial accuracy. The analysis revealed a simple effect for AOI *F*(3,105) = 18.33, *p* < .001, $\eta_{P}^{2}$ = .34, and interaction effects between trial accuracy and AOI *F*(3,105) = 8.35, *p* < .001, $\eta_{P}^{2}$ = .19, between time slice and AOI *F*(6,210) = 3.37, *p* < .01, $\eta_{P}^{2}$ = .09, between trail accuracy, generalization distance and AOI *F*(3,105) = 3.03, *p* < .05, $\eta_{P}^{2}$ = .08 and finally between trail accuracy, time slice and AOI *F*(6,210) = 16.21, *p* < .001, $\eta_{P}^{2}$ = .32. In this analysis the differences between data from correct and error trials were of interest. We ran post hoc Tukey analyses, and such differences were revealed between proportion of fixation times to the taxonomic item in the end of trials in both the interaction between trial accuracy and AOI (*p_Tukey_* < .05,) and the interaction between trial accuracy, time slice and AOI (*p_Tukey_* < .001). In both cases proportion of fixation times to the taxonomic item were higher in trials that led to correct answers than in trails that led to errors (i.e., choice of one of the distractors): *M_Correct_* = and , *M_Error_* = and .

We ran a four-way ANOVA on the log of the number of switches from all trials from six year old children, with trial accuracy (correct, error), generalization distance (near, distant), time slice (beginning, middle, end) and switch type (LL, LTh, LTa, LP, ThTaP) as within factors. Here, we focus on interactions with trial accuracy. The analysis revealed simple effects of time slice *F*(2,52) = 18.12, *p* < .001, $\eta_{P}^{2}$ = .39 and of switch type *F*(4,112) = 15.05, *p* < .001, $\eta_{P}^{2}$ = .35. Results also revealed interaction effects between time slice and switch type *F*(8,224) = 3.05, *p* < .01, $\eta_{P}^{2}$ = .10, an interaction between trial accuracy and time slice *F*(2,52) = 6.02, *p* < .01, $\eta_{P}^{2}$ = .18, and an interaction between trial accuracy and generalization distance *F*(1,28) = 7.71, *p* < .05, $\eta_{P}^{2}$ = .22. Finally, results revealed an interaction between trial accuracy, time slice and switch type *F*(8,224) = 2.53, *p* < .05, $\eta_{P}^{2}$ = .08. However, post hoc analyses (contrasts) did not reveal any significant differences between correct and error profiles.
