# Supplementary material for: How children generalize novel nouns: An eye-tracking analysis of their generalization strategies
Source: PLoS One. 2024 Apr 3;19(4):e0296841. doi: 10.1371/journal.pone.0296841 (PMC10990231; doi:10.1371/journal.pone.0296841)
Supplement: Attachment — Submitted filename: response_reviewers_HowChilrdrenGeneralize .docx [file pone.0296841.s009.docx]

**Manuscript number:** **PONE-D-22-35572**

**Title:** How children generalize novel nouns: an eye-tracking analysis of their generalization strategies.

**Authors:** Eleanor Stansbury, Arnaud Witt, Patrick Bard, Jean-Pierre Thibaut.

Dear Editor,

Thank you for your reply to our manuscript and for your comments. We would also like to thank both reviewers for their insightful comments. Overall, Reviewer 1 and 2 rightfully highlighted that the manuscript would benefit from being positioned in a broader framework. Reviewer 2 also commented in detail on the clarity and precision of the manuscript. We have addressed all of the issues raised by the two reviewers and hope that the manuscript now meets PLOS ONE’s publication criteria.

**General editing remarks**

1. Please ensure that your manuscript meets PLOS ONE's style requirements, including those for file naming

Answer: We have checked and corrected the manuscript’s style and file formats.

2. Please ensure that you provide the correct grant numbers for the awards you received for your study in the ‘Funding Information’ section.

Answer: The “Funding information” section has been amended and the grant from the Conseil regional Bourgogne Franche Comté has been added.

3. Upon re-submitting your revised manuscript, please upload your study’s minimal underlying data set as either Supporting Information files or to a stable, public repository and include the relevant

Answer: Data has been uploaded to the OSF platform and can be accessed via this link: <https://osf.io/hwg7y/?view_only=c444830014ee4c94bb2cdc3f14943b13>. Data has also been added as a supplementary material file.

4. Please amend either the title on the online submission form (via Edit Submission) or the title in the manuscript so that they are identical.

Answer: The title has been amended on the online submission to: How children generalize novel nouns: An eye tracking analysis of their generalization strategies

5. We note that you have referenced [ie. Thibaut, J. P. (1991)] which has currently not yet been accepted for publication. Please remove this from your References and amend this to state in the body of your manuscript: (ie “Thibaut, J. P.[Unpublished]”)

Answer: We have removed this reference from the manuscript and replaced it with a more recent reference: Thibaut, J.-P., & Witt, A. (2023). Children’s generalization of novel names in comparison settings: The role of semantic distance during learning and at test. *Journal of Experimental Child Psychology*, *234*, 105704. https://doi.org/10.1016/j.jecp.2023.105704

6. Please include a separate caption for each figure in your manuscript.

Answer: Captions have been added.

7. Please include captions for your Supporting Information files at the end of your manuscript, and update any in-text citations to match accordingly.

Answer: Captions and in-text citations have been added.

**Response to reviewers**

**Reviewer # 1**

The question of how children explore and categorize is an important one with implications for multiple research areas. The current approach is also innovative and has potential to inform our understanding of children’s generalization abilities, especially because it integrates multiple variables. I was excited to read the study because of its potential. However, that excitement was dampened for a number of reasons. First, the current manuscript is written for a specific niche group of researchers – those that align with the alignment/comparison framework and come from the Gentner/Thibaut school of thought. Because of this, the impact of the current study is diminished and the manuscript would benefit from broader framing and wider inclusion of both theoretical and empirical literature. At the same time, better operationalization of some of the concepts (e.g. how is “aligning” operationalized, how is “semantic distance” measured) is also needed as are missing details in the methods and rationale for the analytic decisions. Overall, the data and question are interesting and relevant, but the particular framing, methods used, and conclusion drawn do not make that relevance clear or broadly applicable. Specific comments for each section are detailed below.

Answer: We agree that the framework could be broader and thank the reviewer for this comment and his/her input in this direction. We now develop the problem more broadly, referring to the experimental literature on lexical biases. See lines 48-73 for example.

As for the question of operationalization of alignment and conceptual distance (called conceptual distance in the new version), we now provide a clearer definition of conceptual distance (see lines 687ss). We understand it as an umbrella term which, in the literature (e.g., Gentner and colleagues), refers to processes that are involved in the comparison of the learning items and how they are compared to the options. In the structure mapping theory of analogical reasoning (e.g., Gentner 1983), it refers to the search for/discovery of structures common to both the source and target domains. In the present study, it refers to the objects to be compared. Our main aim is to better describe the time course of this alignment (as in Thibaut and French, 2016; or French et al., 2017, BRM) by using eye-tracking data to reveal the temporal organization of the search involved during alignment. We have added a paragraph (lines 67 – 69) specifying that “the most important aspect of the process underpinning children’s word generalization is that they come to pay attention to specific dimensions unifying the category (here, object categories)”

Reviewer #1: The introduction is a bit disjointed and some of the theoretical background is long-winded without a clear connection to the current study. For instance, the section on analogical reasoning (beginning end of page 5) assumes the reader is intimately familiar with how analogical reasoning and its methods are different from other generalization/categorization approaches described before.

Answer: We apologize for this lack of clarity. We have tried to provide a clearer explanation and made the connection between analogy tasks and our comparison tasks more explicit in the following paragraphs line 168 - 181: “

In analogy tasks, children must consider a setting in which two items – A and B – are related to each other by a specific relation. This setting is often referred to as the “source domain”. Then, in a second, so-called “target domain” that includes an item – C - , children must select an item – D – such that D is to C what B was to A.

Novel noun generalization tasks and analogy tasks are similar since participants must (a) consider the source of information on the initial items (i.e., learning items in novel noun generalization tasks, source domain items in analogical reasoning tasks) in order to extract commonalities; (b) generalize the information they have found to new items (i.e. one item among multiple generalization options in both the novel noun generalization task and the analogical reasoning task). In our novel noun generalization task, children had to find commonalities between the learning items, extract a representation for the given noun and extend it to the available generalization options (or the target domain items in analogical reasoning tasks). Strategies revealed in the analogical reasoning literature may help us interpret the search patterns observed in our comparison and generalization tasks.

”Our aim was also to highlight the way in which analogical reasoning tasks and novel noun generalization tasks may be underpinned by similar processes in order to justify our decision to take strategies observed in analogical reasoning studies as a basis for our hypotheses in the domain of novel noun generalization. To do this, we now indicate that novel noun generalization tasks and analogy tasks are similar since participants must (a) consider the source of information for the initial items (i.e., learning items in novel noun generalization tasks, source domain items in analogical reasoning tasks) in order to extract commonalities; (b) generalize the information they have found to new items (i.e. one item among multiple generalization options in both the novel noun generalization task and the analogical reasoning task). In our novel noun generalization task, children had to find commonalities between the learning items, extract a representation for the given noun and extend it to the available generalization options (or the target domain items in analogical reasoning tasks). Strategies revealed in the analogical reasoning literature may help in the interpretation of search patterns observed in our comparison and generalization tasks.

Reviewer 1#: When the current study is discussed on pages 7-8 in terms of analogical strategies, exactly how “alignment” is being defined or measured or how the researchers will know when a child has “found a common property” is unclear, which makes the relevance of these strategies even less useful.

Answer: Thank you for this comment. This is, of course, an important, but difficult question. We do not think that we, or indeed that anybody, can tell “when a child has found” a common property. The reasoning behind this type of eye tracking approach is that gazes and switches tell when and which stimuli are looked at and which ones are compared over the course of a trial. From this, we reconstruct the history of the search and identify the search “profile”. We categorize it as “strategy x or y” depending on the profile. We agree that in the previous version of the manuscript, the description of the strategies and their behavioral implications was not sufficiently developed. One of the three strategies was not implemented at the behavioral level and led to no prediction. We now provide a more precise description of the three strategies and the way they are manifested in searches (lines 182-200). We have also included predictions that might result from these strategies (lines 259-283), as well as from a strong shape bias (lines 284 – 289).

Reviewer #1: Relatedly, what if the process of categorization is not based on the conceptual ideals analogical reasoning relies on? For instance, on page 5 (line 94), it is noted that “nouns contribute to the alignment process, most likely by inviting one to engage in the comparison process.” (page 5, line 94). While the evidence does suggest that nouns bias children’s attention, there is still much debate about if it actively “invites alignment” or if the process is conceptually driven or lower-level attention-driven (c.f. Smith & Samuelson, 2006).

Answer: We agree that this is an important point. Our general hypothesis is that novel noun generalization strategies can be interpreted in terms of search strategies. We agree that there is an important debate regarding conceptually driven explanations and lower-level attention-driven processes. However, our experimental approach did address this as we were mainly interested in how children find relevant information when two stimuli are available. The comparison process we refer to, for the learning items, might involve both perceptual and conceptual similarities, as both were available. One common explanation of alignment (e.g., Namy and Gentner, 2002, Child Dev) is that children most likely start with perceptual similarities, which might then lead to other comparisons of the learning stimuli which, in turn, might then themselves lead to further explorations and the discovery of deeper similarities. Consequently, both types of similarities are involved. Whether or not the conceptual information children ultimately come up with is perceptually implemented is a difficult issue for which there is no definitive question. Here, again, we try to understand how children proceed in order to acquire the information. We have tried to make this clearer in the manuscript.

Reviewer #1: More inclusion of work highlighting perceptual properties (e.g. shape bias) or empirical work using looking based measures for generalization-relevant tasks (e.g. Yoshida et al., 2019) would help.

Answer: Thank you for this comment. We have added a section lines 54 – 65.

Reviewer #1: The methods are particularly brief and limited on details as well as rationale. Can the authors comment on what extend using 2D vs. 3D stimuli matters as well as how using familiar referents (vs. entirely novel items such as those used in classic shape bias work) might impact the results? It would seem that the former (2D images) limits the use of perceptual features as one does not have access to things such as material or texture.

Answer: This is an important question. We have clarified our view in the following paragraph (lines 361-368) “Items were pictures rather than 3D objects because 2D pictures have most often been used in novel noun generalization studies and because they are better suited for studying children’s strategies on the basis of eye-tracking measures. It might be thought that 2D pictures are not as informative as 3D items in terms of perceptual input. One question was whether the perceptual distractor would attract sufficient attention, especially in the case of familiar stimuli. However, we built on the experiment of Thibaut and Witt (2023), who used the same stimuli in a no-comparison condition. As expected, children selected the perceptual match above chance level, as predicted by a shape bias”.

Reviewer #1: Likewise, the use of known referents would bias individuals to use taxonomic or thematic relations over perceptual ones, given extensive history with those items. In regards to the use of familiar items then, capturing children’s prior experience with those items and knowledge of their real labels would be relevant. The authors note on page 15 that “participants were supposed to know the items” and “percentage of unknown items was very low” but no statistics or details for this specific sample are given.

Answer: We have replaced our sentence with (line 472-473): “Participants knew the items taken from Thibaut & Witt (2017; 2023), who tested 4- and 5-year-old children with these items”

Reviewer #1: More details also need to be provided in the methods in order to replicate the study.

a. For instances, what is the timing for presentation of the stimuli on each trial? Were all 5 pictures shown at once?

Answer: Thank you for bringing this to our attention. Yes, the 5 pictures were shown at once. We now provide more information about the task and pacing of stimulus presentation in the materials and procedure section, lines 439 – 473.

b. At one point did the verbal directive start/end? How did the task direct children’s attention – e.g. the prompt is “This is a buxi”, but how did the child know what “this” referred to? Was it highlighted, pointed to by the experimenter, no directive (so referent might be unclear), etc.?

Answer: We now clarify this point in the procedure section: lines 439 – 449: “It was critical to limit external elements that might have influenced the children’s gaze patterns during the task (e.g., hand movements by the experimenter, sequential item appearance on the screen etc.). To eliminate the effect of the item’s appearance, all of the trial’s stimuli always appeared simultaneously on the screen, thus making stimulus location (learning and option items) predictable. When the items appeared, the experimenter gave the trial instruction: “*See, Sammy’s mummy* says *this is a buxi*. And *this is a buxi too*. *Sammy must find another buxi. Can you show which one of these three is also a buxi,* *to help Sammy? Can you point to the other buxi?*” To eliminate any effect of the experimenter’s movements, he did not make any hand movements towards the screen during testing. To ensure that this did not affect children’s understanding of the task, a warm-up phase, intended to explain the task to the children, was conducted before eye-tracking recording started”

And lines 450 – 466 : “In the warm-up phase, children saw three trials which were identical to those seen later during the test phase except for the instruction given. In the first warm-up trial, the experimenter added location information and pointing movements to the items he was talking about. “See, Sammy’s mummy says this item at the top of the screen is a buxi. And this one next to it is a buxi too (Exp points to them). Sammy must find another buxi. Can you show which one is also a buxi, to help Sammy? Can you point to the other buxi among these items at the bottom of the screen (pointing at the three options on the screen)?” In the second warm-up trial, the experimenter gave the same instruction without pointing and without location information (e.g., “at the top of the screen”). In the third warm-up trial, the experimenter gave the same instruction as in the test trials, still without pointing. If the child did not answer in the second warm-up trial, the experimenter repeated the instruction together with the pointing movements. It was decided that children who did not answer in the third warm-up trial would be excluded because we could not be certain that they had understood the task. However, no child was excluded at this stage. After the warm-up trials, the experimenter started the eye-tracking calibration phase, which was followed by the experimental trials themselves..”

c. How was the eye-tracking data cleaned and processed?

Answer: Thank you for pointing out the need for these details. They can be found at lines 476 – 478: “Eye-tracking data were recorded as follows. The Tobii 120 eye-tracking device, equipped with two cameras, recorded the position of the two eyes every 8.33ms. Raw data consisted of all spatial coordinates of eye positions throughout trials [74].”

And at lines 559 – 584 “Eye tracking data collection and processing.”

d. Finally, how was a child’s “choice” determined and from what points were reaction time calculated?

Answer: The information can be found in lines 468 - 471: “The child chose an item by pointing to it and the experimenter selected it with the mouse. Response times were recorded from the time when the item appeared on screen to the experimenter’s click, which also ended the trial and cleared the display”.

Reviewer #1: If my interpretation of the results is correct, children switched from L to P more often at the start of the trial – this is entirely consistent with attention-based learning accounts (not just “salience”, line 492). Then, in the middle of the trials, children look to all test items, but by the end of the trials, they are looking more to Ta.

Answer: This is an important and correct remark. We have amended the paragraphs at lines 658 – 672 and 673 – 678 to emphasize this result more clearly.

Reviewer #1: However, knowing at what point the prompt/target word offset in the methods is critical – does the “early” time bin capture pre-word looking or post or both?

Answer: If we understand the reviewer correctly, this refers to the temporal location of the prompt and the point at which we start to take our measurements. The early time bin does capture all gazes and switches produced after the stimuli are displayed, including the pre-word looking. We did not control for this variable. As we say in the methods section, the experimenter specified the noun when the stimulus appeared. The instructions were not pre-recorded and synchronized with the stimuli. The procedure we followed was probably more engaging than a recording, but at the cost of a higher level of variability in the input children received. This is also the procedure that was followed by Thibaut and colleagues with analogies (a more complex and word-intensive task), by Vendetti and colleagues, 2017, as well as by 2018, Vendetti Starr et al, 2017, Frontiers; Starr, Vendetti et al; 2018, Acta psychologica. A pre-recorded session with arrows pointing at the stimuli and a recorded text would have been less variable. In our case, the variability was most likely random and could not have systematically affected the distribution of the gazes or where they started (e.g., as a function of conceptual distance).

Reviewer #1: The analysis approach, on the surface, seems sound, but there is ambiguity as to why specific decisions were made, such as which variables to include or which sub-sample to run the analysis over. The lack of rationale for these decisions make interpreting the results difficult. As one example, both the reaction time analysis and the eye-tracking analysis were only run on trials that led to a correct choice. Was accuracy related to reaction time or looking? Why not run this analysis over all trials?

Answer: Thanks for pointing out this important issue. We now say in the text, lines 480 – 487: “Performance was measured as the percentage of taxonomic generalizations (i.e., percentage of taxonomic choices) and children’s reaction times in milliseconds. The main aim of the present study was to understand the strategies that lead to taxonomic generalization. Following standard practice, we initially focused on correct (i.e., taxonomic answers) trials only, because we wanted to describe search strategies that lead to a correct answer. It is more difficult to interpret incorrect answers as they may result from a real choice or from random choices when children are inattentive (see below for a comparison of high-achieving and low-achieving participants). ”

Please note that we also performed a comparison of the profiles of correct and incorrect trials and that even though there were interactions with this factor, the post-hoc analyses did not reveal any significant “incorrect-correct” difference between any stimuli. The only, and obvious, difference is that gazes were longer for lures in incorrect trials and for the taxonomic match in correct trials. We also address this question in connection with early gazes in our comparison of high and low achievers.

Reviewer #1: There are also some possible inconsistencies (or at the very least, confusion) over results. For instance, on line 387, the authors note that learning distance did not have an effect on generalization, but on line 362, they note that near scores were higher than distance scores, suggesting that distance DID matter. By line 389, the authors note that learning distance and eye-tracking were not related, but no statistical evidence for this is given (and if reaction time was based on looking behavior, lines 381 would contradict this). Thus, it is not clear why learning distance was eliminated from the eye-tracking analysis (line 389), especially when it was one of the key apriori variables...but then again, it looks like learning distance was still included in the analysis (line 425)?

Answer: We would like to remind the reviewer that the study manipulated both the learning distance (i.e., conceptual distance between the two learning items), i.e. a between-factor with two conditions: close, far; and the generalization distance (i.e., the conceptual distance between the learning items and the taxonomic choice among the available generalization options), a within-factor with two conditions: near, distant. Consequently, near and distant relate to generalization distance, not learning distance. We removed the learning distance factor, but not the generalization distance as this was an important factor. We understand that this may have impaired the clarity of the manuscript and have made the terminology clearer.

More precisely, to answer Reviewer #1’s comments:

a. on line 362, they note that near scores were higher than distance scores, suggesting that distance DID matter.

Answer: This is indeed correct for generalization distance, but not learning distance. See current lines 502 - 503: “For 6- and 8-year-old children, an a posteriori Tukey analysis revealed that near generalization scores were significantly higher than distant generalization scores (*p_s_* < .001).”

b. On line 387, the authors note that learning distance did not have an effect on generalization.

Answer: Lines 540 - 544, “Learning distance did not have an effect on the percentage of taxonomic generalization choices and its size effect on reaction times was small. For these reasons, and after verifying that learning distance did not interact with other factors in the eye-tracking analyses, we excluded this factor from further analysis to simplify the design and improve the robustness of our results. Generalization distance was not excluded from the analyses.”

c. line 389, the authors note that learning distance and eye-tracking were not related, but no statistical evidence for this is given (and if reaction time was based on looking behavior, lines 381 would contradict this).

Answer: We have added statistical evidence to support this claim, lines 545 – 557.

d. Thus, it is not clear why learning distance was eliminated from the eye-tracking analysis (line 389), especially when it was one of the key apriori variables...but then again, it looks like learning distance was still included in the analysis (line 425)?

Answer: The analysis, which was previously introduced in line 425, now line 591, includes generalization distance but not learning distance. Indeed, the behavioral data did not show any effect of this factor on generalization performances and had only a small effect on reaction times (see lines 540 – 557 for a full explanation). We therefore decided to remove it from our eye-tracking analysis to increase statistical power. We also reasoned that a non-existent behavioral effect would not lead to strong effects in the eye-tracking analysis, and this was indeed the case.

Reviewer #1: In addition, further information is needed as to why the three-way interaction in looking behavior was dismissed (line 432).

Answer: Reviewer 1 is correct in pointing out the incompleteness of this section. We have amended the text as follows to explain why the interaction in question was disregarded, lines 604 - 607:

Reviewer #1: Finally, the analysis and discussion about low and high achievers seems unsupported, exploratory, and less connected with the rest of the study or analysis. This is particularly true because its not clear that the Learn/Gen ratio is the best way to capture this concept. In general, I would encourage the authors to consider mixed effects models for future work to better capture item and subject differences especially without having to create new composite ratios.

Answer: We have reformulated the introduction to this analysis and also part of the discussion regarding this factor. Please note that significant parts of the discussions have been modified to implement the suggestions of the two reviewers. We have added, line 770 -773, “Following this logic, we tested whether high achievers and low achievers followed the same sequence of gazes at the beginning of the trial in order to confirm (or disconfirm) the importance of early comparisons for correct generalization”

Reviewer #1: Similar to some of the comments on the introduction, there are some assumptions made about the interpretation of children’s behavior that may not be warranted. In particular, switching between the learning items does not necessitate that children are making a conceptually-driven alignment and comparison process. Rather, children may simply be habituating with the stimuli being highlighted. This is consistent with low-level memory and encoding processes and visual processing patterns. Similarly, the structure of the methods directs children to the learning items. Because of this, I would not expect a child to spend more time looking at the 3 test items and thus, support for a “construction” strategy is not likely to arise because of the methodological design. Because of this, I don’t think we can differentiate between the theories/strategies proposed and I don’t think the methods used are completely unbiased to do so. [….] That being said, the data are still potentially interesting (pending details on the analysis as above) and it may be the case that we don’t need to posit one of these alignment strategies in order to inform our understanding of behavioral patterns in comparison tasks.

Answer: This comment is in line with previous comments and thus changes to the manuscript that have been described above. These points are important additions to the manuscript, and we thank Reviewer 1 for highlighting their importance. We have added sections to support this point in the introduction, lines 54 – 65, the results, lines 658– 633, and the discussion, lines 844 – 862, 893 – 905.

Minor points:

- First line of abstract reads a bit awkward and passive. I’m not sure that “comparison setting” is a term that would be familiar to all readers. Also, later in the abstract, the authors refer to learning items and test items, but it’s not clear what the difference is so the brief summary of the results is difficult to interpret.

Answer: This sentence has been rewritten, lines 18 – 22.

- I was surprised to see that the 2002 4-step process view of the shape bias by Smith and colleagues was not cited, nor was work by Oakes and colleagues (e.g. Oakes & Madole) on infant categorization using a similar temporal touching coding

Answer: This process is now described and cited, lines 54 – 65.

- Line 98 – “novel noun generalization” is used in the literature to refer both to novel words with familiar referents (as is the case here), but also novel words with novel referents (such as the shape bias work). Distinguishing which the authors mean is critical.

Answer: “novel noun generalization” refers to novel words with familiar referents and has been clarified in the section from line 78.

- Line 150 – “that the distractors are a priori more salient (see above) than the taxonomic choice”

– it is unclear what “above” refers to or why they are more salient (because prior lit supposes them to be, or that they were experimentally manipulated to be)

Answer: This expression has been removed. The material was tested and results revealing the strength of the relation between learning items and distractors are presented lines 369 – 407.

- Line 331 – it is not clear what this warm-up phase would look like or entail (or why it is helpful) if there is nothing on the screen, but the primary task is behavior (looking) toward the screen.

Answer: The warm-up phase has been clarified lines 450 - 464 to highlight its importance in eliminating any effect of the experimenters’ movements on the eye tracking data (see lines 439 – 449).

- Line 376 – “significantly different” – in what direction? Which group was faster?

Answer: This is now explained lines 513 – 525.

- Line 410-411 – “can be interpreted as an attempt to find a relation between X and Y” – is this universally true and can we be sure of it? Children may be switching gaze not for comparison/relation purposes but because of habituation and boredom with the prior item, e.g. This should be more carefully phrased (as should similar places in the paper).

Answer: Reviewer#1 would be right to make this comment if we had analyzed all of the children’s answers. However, the data was carefully selected to avoid such situations and ensure that children fully completed the task. Indeed, we only selected data from correct trials, thus excluding trials that were presumably not completed correctly (see lines 486 - 490). It is unlikely that children who gave correct answers didn’t understand or concentrate on the task. We also selected robust eye tracking data (see lines 560 – 571), meaning that “inattentive” participants would probably have been excluded. It is also unlikely that children who focused well on the screen were bored. Finally, the search patterns were systematic across ages and levels of difficulty (see lines 979 – 985) while, in the unlikely case of boredom, children would have exhibited unsystematic, random search profiles.

- Please give specific p-values unless it is <.001

Answer: This correction has been made throughout the manuscript.

- It might be useful to have a time course figure showing moment-to-moment (usually 10ms bins) gaze patterns to each item. Such a figure would illuminate both the early-mid-late patterns as well as switches in relation to time.

Answer: Unfortunately, this was difficult to do given the format of the data.

- Line 476-477 – something is off grammatically here (I think there is a repeated “was an interaction” phrase)

Answer: Indeed, “was an interaction” was repeated in the sentence and one occurrence has been removed.

- The main aims listed in the first paragraph of the discussion do not seem to be identical to those listed at the end of the intro. I suggest listing the aims explicitly in both places, using parallel wording and copying that wording throughout the results as well.

Answer: Thank you for this suggestion, which we have implemented. The wording is now identical in the introduction and the discussion.

**Reviewer #2**

Reviewer #2: First, there were many places in the introduction where a clear definition of the task would have been helpful. The authors frequently refer to a “comparison task” and will reference an “analogical task” but do not take the time to adequately summarize either methodology. Given that the present study builds on this prior work, I think offering a clear overview of the critical methods and then using consistent terminology to refer to the tasks would greatly help the reader better understand the greater context of the paper.

Answer: We thank the Reviewer for pointing this out. We have added a paragraph lines 78 – 88 as follows: “Novel noun generalization tasks are commonly used to investigate the way children extend novel names. In such tasks, children are usually shown a learning (otherwise known as a standard or reference) item. Past studies have made use of both pictures of objects and replications of objects. The item is associated with (often) a nonword (e.g. “*This is a buxi*” while showing a bracelet). The children are asked to choose another item among a set of options that could also have the same name (e.g., “Which one is also a *buxi*?” among, for example, a necklace and a tyre in Fig 1.A, below). The specific options from which the children must choose depend on what the researcher wishes to study: one is a “correct” item that is taxonomically related to the learning stimuli (e.g. another piece of jewelry in the present example), while the others are distractors that can be semantically or perceptually related to the learning stimuli [19, 32, 33, 34, 35, 36, 37].”

Reviewer#2: Second, the introduction is heavily focused on the role of alignment in perceptual and conceptual similarity, but it pays little attention to the body of work on noun generalization in early childhood. In particular, I was a bit surprised that there was only a brief mentioning of the Novel Noun Generalization task (and only two references on it), especially since readers of the journal may look at the title of this manuscript and similarly be surprised that that this body of work was not addressed more fully. Here, I think an important contribution of the work shows patterns of noun generalization later in childhood, but the impact is only made once the reader is aware that most previous research on NNG is earlier in childhood.

Answer: We thank Reviewer#2 for this comment on the framework and have accordingly added references and details in the introduction. For example, lines 83-87.

Reviewer #2: I was really surprised that the authors did not have a language measure as a control variable in their models; given the abundant literature showing the role of vocabulary in noun generalization, I think its absence in the present study is worth discussing at some point in either the method or general discussion.

Answer: Thanks for this comment. In fact, we did indeed collect such a measure as part of a broader project. We have reworked the manuscript to incorporate this aspect and added that no correlation between vocabulary and generalization was found, lines 427 - 431: “Children’s general knowledge and language development were tested using the EVIP (Echelle du Vocabulaire en Images de Peabody) standardized French vocabulary test. As expected, children’s scores improved with age, but no significant correlations were found between vocabulary scores and generalization scores (Pearson partial correlation r = 0.07, *p* = 0.45).

”

Reviewer #2 : Third, the authors conclude that the results are consistent with “alignment” but never really offer an alternative theoretical perspective, either in the introduction or discussion. If children are not visually aligning and comparing the stimuli, what else would they be doing? Clarifying the alternative option would strengthen the conclusions drawn, especially in lines 607-608

Answer: Following similar comments made by Reviewer 1#, we have enlarged the framework and updated the introduction and the discussion. We also discuss more options and hypotheses in both the introduction and the discussion. It should be noted that alignment is an umbrella term which is compatible with different interpretations. The main aim of the present contribution is to provide a description of the temporal organization of the task and discuss possible strategies. Cf responses to Reviewer #1.

Reviewer #2: Moreover, I wonder if including a trial-by-trial analysis would provide insight into the different strategies children use. Here, the "first gaze" information is useful, but I could imagine that as children gain familiarity with the task that their first gazes and strategies may change.
Answer: This is an interesting comment. Children had to perform the same task across trials, requiring them to look at the learning items and make comparisons, as shown by the older children’s profiles. It is not impossible that they progressively automatized this search pattern as they proceeded through the experiment. It is also not impossible that the search sequence might have varied slightly across trials and/or participants. However, if the variations had been too great, the regularities we were looking for would not have existed in the averaged data. Nevertheless, we did indeed find such regularities. In other words, our hypotheses were about the order in which, on average, children would direct their first gazes. Even if there had been potential variations in this order, our hypotheses were not intended to deal with this question and the variations would have been difficult to interpret. Although we consider this to be an interesting question, it is beyond the scope of our research.

Reviewer #2: Fourth, though this may be a “minor comment”, I was surprised that the authors did not include the rationale for the ages, especially since 7-year-olds were not included altogether.

Answer: We have added this rationale, lines 220 – 231: “We tested 5-, 6-, and 8-year-old children in the novel noun generalization task. Six- and eight-year-olds were our two main target groups as we knew [37] that the younger group might still have difficulties with the task, since they were young enough to still be attracted to salient distractors available among the generalization options alongside the taxonomic option. At the same time, we also knew that the older group would primarily give taxonomic answers. Thus, eight-year-olds were expected to produce a majority of mature strategies, whereas six-year-olds were expected to produce strategies that would reflect a greater influence of the distractors. We also added a five-year-old group after pretests as we became aware that this age group might yield reliable eye-tracking data (see below) and would also testify to young children’s difficulties in conceptualizing taxonomic relations, given that it is far more difficult to collect eye-tracking data at such a young age and that this data contains a lot of noise (see Thibaut et al. 2016 for the case of analogies).

”

Reviewer #2: Finally, I struggled at times with the overall readability of the paper. The structure of the paragraphs could be hard to follow, especially when laying out the different strategies, discussing the similarity ratings for the stimuli, and reporting the results. Though the approach to the study was novel and interesting to learn about, I think working carefully with a copy editor (in addition to some of the suggestions I make below) would greatly strengthen the readability of the manuscript overall.

Answer: We thank Reviewer #2 for his advice and have reedited the manuscript carefully.

Minor points:

Line 19 – Abrupt start to the abstract. I think it may help the audience to describe the comparison task before discussing results and limitations.

Answer: We have rephrased this initial section.

--Paragraph starting with line 54 – it would help to name the paradigm. It is a “Novel Noun Generalization” task.

Answer: This has been clearly specified.

--Line 59: Not sure what the authors mean by “learning designs”

Answer: For clarity, we have changed the terminology to: novel noun generalization in a comparison setting, or learning setting with comparison.

--Line 61: for “learning stimuli”, do the authors mean two or more objects belonging to the same basic category? I wonder if an example would clarify what they mean, as the sentence is hard to understand.

Answer: For clarity, a figure and examples that reference it have been added.

--Line 64: Genter and Namy’s (1999)

Answer: The correction has been made, thank you.

--Line 65: I suggest replacing “imaginary” with “novel”

Answer: The correction has been made, thank you.

--Line 69: The authors mention a “learning phase” but do not explain what happens during the phase; I think clarifying this point would also help distinguish what Gentner and Namy found in the no-comparison condition

Answer: The correction has been made, thank you. See lines 78-82.

--Lines 79 and 80: eliminate “would”

Answer: The correction has been made, thank you.

--Line 80: replace “neglect” with “ignore”

Answer: The correction has been made, thank you.

--Line 81: “authors” is ambiguous

Answer: The correction has been made, thank you.

--Line 81-82: Getting a little lost on the different paradigms. I think a clear description of each would be really helpful, especially as the authors continue to refer to them.

Answer: For clarity, the explanation has been split into two separate paragraphs dealing with analogical reasoning tasks and then novel noun generalization tasks, respectively. See lines 165 - 178. Both paragraphs have been rephrased.

--Line 89: Unsure what the authors mean by “mapping” -> what are individuals mapping? Labels?

Answers: This has been rephrased lines 125 – 132.

--Line 98: remove “the”

Answer: The correction has been made, thank you.

--Line 109: Hard to follow; again, I think a clear definition of the terminology would be very helpful

Answer: The correction has been made, thank you.

--Line 114-115: Consider defining “source domain” and “generalization-target domain”. For this paragraph, I would consider adding a figure to depict the different combinations of strategies.

Answer: We have rephrased the paragraph lines 169 – 178 as follows: “

Novel noun generalization tasks and analogy tasks are similar since participants must (a) consider the source of information on the initial items (i.e., learning items in novel noun generalization tasks, source domain items in analogical reasoning tasks) in order to extract commonalities; (b) generalize the information they have found to new items (i.e. one item among multiple generalization options in both the novel noun generalization task and the analogical reasoning task). In our novel noun generalization task, children had to find commonalities between the learning items, extract a representation for the given noun and extend it to the available generalization options (or the target domain items in analogical reasoning tasks). Strategies revealed in the analogical reasoning literature may help us interpret the search patterns observed in our comparison and generalization tasks.”

--128-131 -> hard to follow. I wonder if a figure for previous paragraph would also facilitate discussion of strategies in this paragraph

Answer: A Figure has been added line 89.

--134 -> Not sure I understand how the Task goal strategy differs from previous strategies. Are these all strategies used within the same paradigm? Is a “task goal” strategy something that can be addressed by trial-by-trial analyses of the task?

Answer: Details have been specified lines 193 – 200.

--177: Wonder if a discussion of semantic distance earlier in introduction is warranted to provide motivation for this manipulation

Answer: We now discuss this aspect earlier in the manuscript, see lines 232 – 251.

--249: replace “&” with “and”; remove comma after “Witt”

Answer: The correction has been made, thank you.

--265: “Students” = what age? Who normed them? Overall, I wonder if a description of the similarity ratings is best saved for the supplemental materials. At present, it distracts from the focus of the paper.

Answer: The following details have been added lines 369 - 370: “mainly psychology students, who participated in return for course credits, mean age = 20.15 years; 47 females”.

--319: replace common with semi-colon

Answer: The correction has been made, thank you.

--330: move period inside quotation marks

Answer: The correction has been made, thank you.

--331: did not understand what happened during the warm-up trial

Answer: The description of the warm-up trial has been made clearer

--341: Replace “randomly assigned” with “randomized”

Answer: The correction has been made, thank you.

--344: Did they prescreen children’s knowledge of items? What was “very low”? Were trials dropped if children did not know the items?

Answer: This line has been replaced by: “We used fourteen familiar categories which were adapted from Thibaut and Witt (2017) (see S1 Table for a full list of materials).” Line 339 – 340.

--364-5: Report t-values

Answer: The correction has been made, thank you.

--393: “was” with “were”

Answer: The correction has been made, thank you.

--476-477: repetition of “interaction”

Answer: The correction has been made, thank you.

--502: The work on the first gaze makes me wonder if first gaze aligns with hearing the label and if first gaze changed across trials as children became more familiar with the task

Answer: cf previous answer to the comment about the usefulness of "first gaze" information. However, I could imagine that children’s first gazes and strategies may change as they become familiar with the task.

--548: Achievers -> need to define this sooner instead of “see below”; is there precedent for this approach?

Answer: The correction has been made, thank you.

--609 – fixation (remove “s”)

Answer: The correction has been made, thank you.

--610 – generalization options? I think consistent labeling would be very helpful

Answer: The correction has been made, “generalization options” is used throughout the manuscript to replace “generalization items” for example.

--615-20 – a bit hard to follow

Answer: The paragraph has been rephrased, thank you.

--623 = is -> are

Answer: The correction has been made, thank you.

--626 = remove “which is the pattern we found” as it is redundant

Answer: The correction has been made, thank you.

--Paragraph 621 = watch for redundancy

Answer: The paragraph has been checked and corrected, thank you.

--637 = I found myself asking “how do you know?”
--641 = “ration” => “ratio”

Answer: The correction has been made, thank you.

--659-661 = hard to follow

Answer: The paragraph has been modified.

--664 = not sure what the authors mean by control

Answer: This section has been modified.

--704 = “problems” -> “problem”

Answer: The correction has been made, thank you.
