# Supplementary material for: How children generalize novel nouns: An eye-tracking analysis of their generalization strategies
Source: PLoS One. 2024 Apr 3;19(4):e0296841. doi: 10.1371/journal.pone.0296841 (PMC10990231; doi:10.1371/journal.pone.0296841)
Supplement: Attachment — Submitted filename: Response to reviewers.docx [file pone.0296841.s010.docx]

**Manuscript number:** **PONE-D-22-35572R1**

**Title:** How children generalize novel nouns: an eye-tracking analysis of their generalization strategies.

**Authors:** Eleanor Stansbury, Arnaud Witt, Patrick Bard, Jean-Pierre Thibaut.

Dear Editor,

Thank you for your reply to our manuscript and for your comments. We would also like to thank both reviewers for their insightful comments. Overall, Reviewer 1 and 2 rightfully highlighted that the manuscript would benefit from a more detailed report of the methodological decisions taken throughout the study. We feel that addressing these aspects has helped strengthen the manuscript and make it more accessible for future research. We have addressed all of the issues raised by the two reviewers and hope that the manuscript now meets PLOS ONE’s publication criteria.

**General editing remarks**

Please review your reference list to ensure that it is complete and correct.

Answer: We have corrected mistakes in the reference list.

**Response to reviewers**

**Reviewer # 1**

Reviewer 1: The discussion of analogical reasoning and parallels with that task are less clear – while both analogical reasoning and noun generalization tasks require comparison and generalization, the analogical reasoning task by design is a much more cognitive complex task and requires a much higher level of logistical processing. The “A is to B as C is to D” comparison is very difficult for young children to do, meaning the ages the tasks are used with also vary. By trying to draw analogical reasoning literature into the current study just makes the current study hypotheses more convoluted and posits processes that don’t need to be proposed in order to examine looking behavior in generalization tasks. Similarly, by line 267, there are hypotheses being proposed that directly tie to the strategies, but since the task here is not an analogical reasoning task, it is unclear how those might apply or if they would even be relevant.

Answer: We thank reviewer 1 for highlighting this paragraph that we agree needed improvement to be clearer. We have a double aim here, make the paragraph as small and clear as possible to not distract the reader from the general line of reasoning – in line with reviewer 1’s comment -, while still justifying why we consider analogical reasoning strategies as an inspiration for our generalization strategies. This meant to soften the parallel between analogical reasoning and our task, and the idea that the two tasks were similar. We know say that we borrow from the analogical reasoning literature, because there are similarities, while acknowledging differences. To fit both aims and thus answer Reviewer 1’s remarque, we rephrased the paragraph in a clearer manner, as follows: “In the following section, we borrow our candidate strategies for the lexical comparison task from the analogical reasoning literature. Both tasks are generalization tasks in which participants have to generalize properties from a base domain to a target domain. Even though the two tasks are of a different level of complexity, both are alignment tasks between domains [60, 61, 62]. In analogy tasks, the setting shows two items– A and B – (the source domain) which are connected by a specific relation. Then, the target domain is introduced with an item – C - , and children have to select an item – D – that goes with C in the same way B goes with A. At a general level, both our noun generalization task and the analogical reasoning task request (1) the analysis of the source items (i.e., learning items and -A B- items) in order to extract commonalities; (b) generalize the information they have found to new items (i.e. one item among multiple options in both tasks).”

Reviewer 1: There are some remaining questions and possible concerns about decisions in the methods and analyses as well. First, on Line 330 – choosing a sample size simply because a prior study did is not a valid rationale for power. While the authors may not have conducted an a prior power analysis to determine the sample size, post hoc inclusion of power that confirm that this sample is sufficient to detect the effects anticipated is needed. This is especially true because of the large models needed to analyze the looking behavior.

Answer: We thank Reviewer 1 for insisting on the precision needed here. We have added l. 333 – 347, the details of the a priori power analysis based on results from the studies that entailed the present work: Thibaut & Witt (2023) for generalization data and Thibaut & French (2016) for eye-tracking data.

Reviewer 1: line 545 indicates that post-hoc, a key variable was removed from the analysis because it wasn’t significant. Yet learning distance seemed to be a critical element of the design and hypotheses, thus removing it simply to “improve robustness” of the results sounds an awful lot like p-hacking. I understand if there is a better model fit without it, but then more details must then be presented demonstrating that patterns of significance do not change and the full planned analyses need to be presented at least in the supplement.

Answer: We thank Reviewer 1 for this comment and have added the full analyses to supplementary materials (see S3). We have also rectified the phrasing of this section in the manuscript (see l.604-624) to explain better our choice in pursuing analyses without learning distance. The main argument was that the interaction effects involving learning distance (for both proportion of fixation times and log of the number of switches) did not reveal significant differences between learning groups as revealed by post hoc Tukey analyses.

Reviewer 1: Another concern about the data is that 19 children were dropped for not having good calibration and additional 32 for poor tracking. While the sample size is large, data needs to be presented that this significant drop in participants did not impact the overall power.

Answer: The sample size was much larger than the sample size indicated by the a priori power analysis. Moreover, post hoc power analyses on the most important interactions with eye tracking data show good power results. Indeed, in the case if proportion of fixation times a post hoc power analysis run for the interaction between time slice and AOI revealed a power = 1.00. In the case of log of the number of switches the post hoc power analysis on the interaction between time slice and switch type revealed a power = 1.00. Thus, results can be considered robust even though the final sample lost participants.

**Minor points:**
- Citation 21 listed on line 27 and 78 doesn’t seem to match the argument. From what I can tell, citation 21 is not about the shape bias.

Answer: Citation 21 has been changed from Kucker et al. 2018 to Kucker et al. 2019 that does address shape bias.

- Line 88 – the taxonomic choice won’t always be the “correct” item – which is correct depends on the task, objects available, semantic context, etc. Either correct this sentence to specify that in this specific example in figure A, taxonomic is most likely correct, or better yet, just remove the word “correct”.

A similar issue to this comes up on page 10 (lines 219) when the authors presume that the taxonomic is the correct answer. While the authors may hypothesize that taxonomic may be the more frequent response and they may analyze “proportion of choices to taxonomic choice”, assuming that it is the “correct” answer for a child is overstepping what we can know about children a priori. Being more careful with the wording used is important here.

Answer: We now explain why and in what terms the taxonomic choice can be considered as the “correct” answer depends on the task. Here we use “correct” for the taxonomic item and have explained this choice l. 89 – 94.

- Line 229 – need a citation for the fact that 5-year olds are distracted by salience (and that it is actually distraction vs. immature categorization abilities)

Answer: Thank you, we have added the reference Gelman & Meyer (2011), l. 231.

- Line 331 – define “some”

Answer: A paragraph was added l. 332 – 351 to specify a priori sample size power analyses. The amount of data to be lost was estimated and figures were given here enabling us to delete “some”.

- Line 346 – which “learning items” are you referring to here? It would seem as though both the curb chain and the watch are both perceptually and conceptually similar to the bracelet, but the curb chain is much more perceptually similar and watch a bit less so.

Answer: Learning items have been specified l. 360.

- Please give specific p-values when available

Answer: Specific p-values have been given for all p > .01.

- The high vs. low achieving analyses need to be more clearly outlined as exploratory as there was no a priori plan for this nor hypotheses until after the data were collected.

Answer: This exploratory aspect of the high vs low achiever analyses has been specified l.837 - 839.

**Reviewer # 2**

Reviewer 2: I noticed that the authors elaborated more fully on the rationale for 2D as opposed to 3D stimuli (line 362) in the revised manuscript, but the rationale presented seemed questionable. In particular, many iterations of the NNG task with toddlers have been done with 3D objects (e.g., Landau et al., 1998; Samuelson & Smith, 2002; Perry et al., 2010), and there has been a surge in the use of head-mounted eye-trackers to better understand the real-time visual dynamics of children's interactions with objects, used with children as young as 18 months of age (e.g., Yoshida & Smith, 2008; Yu & Smith, 2012). I think it would be beneficial to further clarify why 2D objects were a particular strength (or necessity) of this study's design.

Answer: Thank you. There are studies with 3D objects and eye tracking studies can be done with 3D objects. No doubt about this. We changed the text accordingly. Most of the studies with comparison, however, have been performed with pictures or even drawings of existing objects (see Gentner and colleagues contributions, or Thibaut and colleagues, Xu Tennenbaum 2007, a b; Spencer Smith et al. 2011) with only a few exceptions. In the lexical learning literature, more broadly, many studies rely on artificial stimuli, drawings, pictures. However, we concur that many other studies were also built around 3D objects. In most of them, the overarching question was targeted at which dimension(s) of the stimuli would influence children's extension the most. In this case, it is crucial to build stimuli with the dimensions which would realistically mimic the dimension availability. Hence 3D objects. In most other experimental situations realistic 2D pictures afford all the information children need to identify and discriminate the objects, shape, color and texture, which was the case here. In this context, our quest for children's strategies was required this type of stimuli and in a design in which stimulus location would be easy to control. Given that our former studies have shown that our stimuli could be used to assess the role of comparison, they did a perfect job here. Note that 3D toys are not immune to problems either when they are not made of the same materials as their real-world counterpart.

Reviewer 2: I think there needs to be further clarification on the use of familiar objects. Although the authors mention that these categories have been used in similar studies before, I think the conclusions drawn from the study's results may be significantly impacted by the fact that children already have labels for the objects and categories. Additional rationale and/or discussion on the use of familiar as opposed to novel stimuli (as is seen in many other word learning studies) and how it may have impacted the results should be included in the manuscript.

Answer: We added a justification in the manuscript (l. 376 - 384) the impact of the objects familiarity, namely the impact of the labels children already have for the objects may be questioned. More broadly, the question of the impact of children’s prior knowledge is addressed by this comment. We have added three arguments suggesting that children’s word generalizations are not driven by children’s knowledge even though the material used is familiar to them.

Reviewer 2: It would be beneficial to more clearly define the different time bins. In a recently published study, Bakopoulou, M., Lorenz, M. G., Forbes, S. H., Tremlin, R., Bates, J., & Samuelson, L. K. (2023). Vocabulary and automatic attention: The relation between novel words and gaze dynamics in noun generalization. *Developmental Science*, e13399. **)** illustrated that the presentation of the novel noun shifted children's attention in a novel noun generalization task, especially for children who displayed a strong shape bias. Knowing that the timing of the prompt impacted the cascade of looks in a younger age group, it seems plausible that how the bins were defined could significantly impact the interpretation of the results in the present study.

Answer: Thanks for mentioning this reference. In the present study, time bins were defined (i.e., in three equal time slices according to the trial’s total time) and thus vary across trials and participants. For this reason, the position of the novel word and the gaze profiles is not controlled across trials. In the case of Bakopoulo’s et al. (2023), that investigates the relation between the word and the gaze profiles, defining the time bins as was done here would have been a significant impediment to the study. In our case, however, were the question was the investigation and qualification of the overall strategy profile, this a minor limit of the work. Especially considering that result interpretations address strategie’s general profile and how they can be qualified referring to existing models, rather than the relations between profiles in different times slices, which may indeed be affected by how time bins were defined.

Reviewer 2: Finally, I found myself questioning why the authors would restrict their analyses to trials with only a "correct" taxonomic match. In particular, as I read lines 632-633, I was not surprised by the results because we know that individuals are likely going to turn their attention to the object they plan to select so as to guide their reach (or point, in this case). It made me wonder if the pattern of looking behavior was different for trials with errors, perhaps especially for the low generalizers. Moreover, though the authors shared in their response that they were interested in average looking behaviors across the 14 trials, I was still left wondering if overall attention to the distractor items decreased across trials, especially for children who were good at generalization.

Answer: As Reviewer 2 mentions this question about differences between “correct” strategies and “error” strategies arises when reading the manuscript. First, we can remind reviewers that the question here considered specifically taxonomic generalization strategies, and thus solely the generalization strategies that led to taxonomic or “correct” answers. This is the main argument to explain that eye tracking data from trials that led to correct answers only was considered. However, this does not undermine the interest that arises about “error” strategies. Therefore, we have added a reference to the analyses comparing correct and error eye tracking data l. 727 and the full analysis in supplementary materials. As the analyses show, very few differences are found between eye tracking data from correct and error trials. Only fixation times to the taxonomic item change at the end of trials, these intuitively are longer in correct trails than error trials were participants chose one of the distractors. As for the attention to distractors across trials, the formatting of the data in the present study renders the question too difficult to answer at present.

**Minor points:**

--The revised abstract could be hard to understand at times. In particular, I think it would be beneficial to more clearly define what is meant by learning items and to more clearly delineate between the different generalization options. In it's current form, the organization of the options is quite hard to track; making these examples clearer is critical, especially given the framing of the study.

Answer: The abstract has been corrected and clarified.

--line 19: favor (not favors)

Answer: Thank you the correction has been made l. 19.

--line 31: I recommend using a semi-colon instead of "and"

Answer: Thank you the correction has been made l. 28.

--line 97: Should revise to say "single-learning item"; the current phrasing suggests that participants see only the learning item before the test items (which would heavily demand working memory), whereas most often the learning and generalization items are presented simultaneously. The novel aspect of this study is to have multiple learning items, so revising the language here will be helpful.

Answer: The sentences has been changed and a clarification sentence has been added l. 84.

--line 159: remove the comma before the question mark

Answer: thank you the correction has been made.

--line 170: I think removing "and they have been described in previous studies" would improve the clarity of the writing

Answer: thank you this has been removed.

--line 189: "has" instead of "have"

Answer: thank you the correction has been made l. 167.

--line 209: hard to follow; consider revising, perhaps to something more in line with discussing the real-time developmental differences in processing during the task

Answer: thank you, we have rephrased and added precisions in brackets l. 213 – 220.

--line 235: I appreciate that the authors added a rationale for the ages, but I struggled to understand the rationale for 5-year-olds here, especially knowing that eye-tracking studies have been conducted with much younger children.

Answer: It is true that eye tracking data came be collected for children under five years. However, combining the difficulty of the tasks used here, their length and eye tracking data collection is too ambitious. It can be noted that the task was tested with four-year-old children but over 75% of the data was lost.

--overall, the use of the word "aims" appears frequently in the introduction. Given the 5 aims that appear in the intro and discussion, the authors should examine the other places where they use the word "aim" to see if a different word would be more appropriate

Answer: We have kept the word “aim” when referring to the 5 aims and have used synonyms everywhere else, for example l. 264.

--line 395: generalization item"s"

Answer: thank you the correction has been made.

--line 395-402: I had a hard time interpreting the results as presented here.

Answer: the sentence has been slightly rectified to avoid the ambiguity that led to difficult comprehension

--all p-values should have the exact value reported unless less than .01

Answer: specific p-values have been given for all p > .01.

--line 496: Did the authors mean to include a main effect of age as well? The statistics are reported for it in line 497.

Answer: the sentence has been slightly rectified to avoid the ambiguity that led to difficult comprehension

--lines 508-512: I appreciate the detailed reporting of the t-tests but recommend revisiting the prose, as organization here made results a little hard to interpret.

Answer: the sentence has been slightly rectified to avoid the ambiguity that led to difficult comprehension

--line 543: effect (not size effect)

Answer: thank you the correction has been made.

--line 546: double negative hard to follow; perhaps instead say that since generalization distance interacted significantly with performance, it was included in the remaining analyses

Answer: thank you the correction has been made.

--line 584: This paragraph should be moved up to earlier in the results section so as to clarify the corrections used throughout the analyses

Answer: thank you the paragraph has been moved to the beginning of the section

--lines 590-591: slightly repetitive

Answer: thank you the sentences has been made less repetitive

--line 593: Because our sample size was large, we…

Answer: thank you the correction has been made.

--line 612: analyses

Answer: thank you the correction has been made.

--lines 613-614: need to clarify the contrasts of interest

Answer: thank you the correction has been made.

--lines 685 & 686: comma after end

Answer: thank you the correction has been made.

--line 896: seems like a word is missing

Answer: thank you the word has been added
